# Human serum preβ1-high density lipoprotein levels are independently and negatively associated with coronary artery diseases

**DOI:** 10.1186/s12986-016-0093-y

**Published:** 2016-05-17

**Authors:** Yunqin Chen, Jibin Dong, Xueying Chen, Hui Jiang, Ahmed Bakillah, Xiaojin Zhang, Zhiqiang Li, Jia Yin, Donghui Liang, Yunzeng Zou, Mahmood Hussain, Marina Cuchel, Daniel Rader, Haozhu Chen, Junbo Ge, Xian-Cheng Jiang

**Affiliations:** Shanghai Institute of Cardiovascular Diseases, Zhongshan Hospital, Fudan University, Shanghai, China; Department of Cell Biology, SUNY Downstate Medical Center, 450 Clarkson Ave, Box 5, Brooklyn, NY 11203 USA; School of Pharmacy, Fudan University, Shanghai, China; Obstetrics & Gynecology Hospital, Fudan University, Shanghai, China; Southern Medical University, Guangzhou, China; Division of Translational Medicine and Human Genetics, Perelman School of Medicine, University of Pennsylvania, Philadelphia, USA

**Keywords:** preβ1-high density lipoprotein, Native PAGE system, Coronary artery diseases

## Abstract

**Background:**

Serum preβ1-high density lipoprotein (preβ1-HDL) was defined by two-dimensional non-denaturing linear gel electrophoresis and apolipoprotein A-I immuno-blotting. Serum preβ1-HDL seems to play an important role in reverse cholesterol transport, a well-known anti-atherosclerosis process. However, there are still debatable questions for its quantification and coronary artery disease (CAD) relevance.

**Methods:**

We isolated the preβ1-HDL using a new native polyacrylamide gel electrophoresis (PAGE) system and lipid pre-staining serum. We established a two-demensional gel electrophoresis system.

**Results:**

We measured the preβ1-HDL in Tangier disease patients and subjects with cholesterol ester transfer protein (CETP) mutation. The preβ1-HDL is clearly separated from lipid-free apoA-I monomer and cannot be converted into other HDL particles under lecithin-cholesterol acyltransferase (LCAT) inhibition. This preβ1-HDL is a spheroidal particle with the highest apoA-1/cholesterol ratio and highest density (≥1.21 g/ml), as compared with all other HDLs. Importantly, we found that serum from subjects with Tangier disease or with cholesterol ester transfer protein (CETP) mutation have no detectible preβ1-HDL particles. We recruited a total of 102 subjects underwent diagnostic coronary angiography and measured their preβ1-HDL levels. Among them, 56 had no stenosis of coronary artery and 46 were diagnosed as CAD, which was predefined as the presence of a luminal diameter stenosis ≥50 % in at least 1 major coronary artery territory. We found that preβ1-HDL is independently and negatively associated with the severity of the coronary artery stenosis (Gensini score).

**Conclusion:**

We established a novel and simple method for human serum preβ1-HDL quantification. We found that human lower preβ1-HDL is an independent predictor for severer coronary artery stenosis.

## Background

HDL cholesterol concentration in the blood is inversely proportional to coronary artery disease (CAD) risk [1]. This relationship is thought to be mediated by the ability of HDL to transport excess cholesterol from peripheral tissues back to the liver for excretion, a process known as reverse cholesterol transport [2]. An understanding of the molecular events in HDL formation is thus prerequisite for the development of therapeutic strategies to raise HDL cholesterol levels and protect against atherosclerosis. However, recent research has indicated that HDL particles are very heterogeneous, and indeed increasing total HDL cholesterol does not reduce CAD risk [3–8]. Thus, it is important to characterize each HDL particle subclass in terms of its pro- or anti-atherogenicity.

There are many subclasses of HDL depending on the analytical technique used for separation. HDL can be separated from non-HDL by chemical precipitation methods [9–11]. Likewise, HDL can be separated into HDL_2_ and HDL_3_ by ultracentrifugation [12, 13]. HDL particle size distribution can also be assessed by native PAGE [14, 15]. Two-dimensional nondenaturing linear gel electrophoresis, immunoblotting, and image analysis were first utilized by Castro and Fielding in 1988 to describe preβ1-HDL, preβ2-HDL, and αHDL [16]. Although free apoA-I was not seen on the two-dimensional gel in that first paper [16], Francone et al. from the same lab clearly showed that lipid-free apoA-I could be separated from preβ1-HDL [17]. In 1993, Asztalos et al. used a modified two-dimensional gel system to define 12 subfractions of HDL, including preβ1-HDL and preβ2-HDL, but they did not indicate whether the preβ1-HDL they identified contained lipid-free apoA-I or not [18]. Since then, the same method has been utilized in many clinical studies, including those concerning CAD patients, and the results suggest that an elevated level of preβ1-HDL is a risk factor for CAD [19–21]. The same method was used to show that subjects with Tangier disease still have preβ1-HDL but that HDL maturation is impaired [22]. Miyazaki et al. very recently showed that in their hand the so-called “preβ1-HDL” is, in fact, lipid-free monomolecular apoA-I [23]. Thus, it is necessary to re-valuate how preβ1-HDL is defined and measured. In this study, we established a direct measurement for preβ1-HDL in human serum. Moreover, we found no detectible preβ1-HDL in Tangier patients and in the subjects with cholesterol ester transfer protein (CETP) mutation. Importantly, preβ1-HDL is reduced in CAD patients.

## Methods

### A new native polyacrylamide gel electrophoresis (PAGE) system

**Fig. 1:**
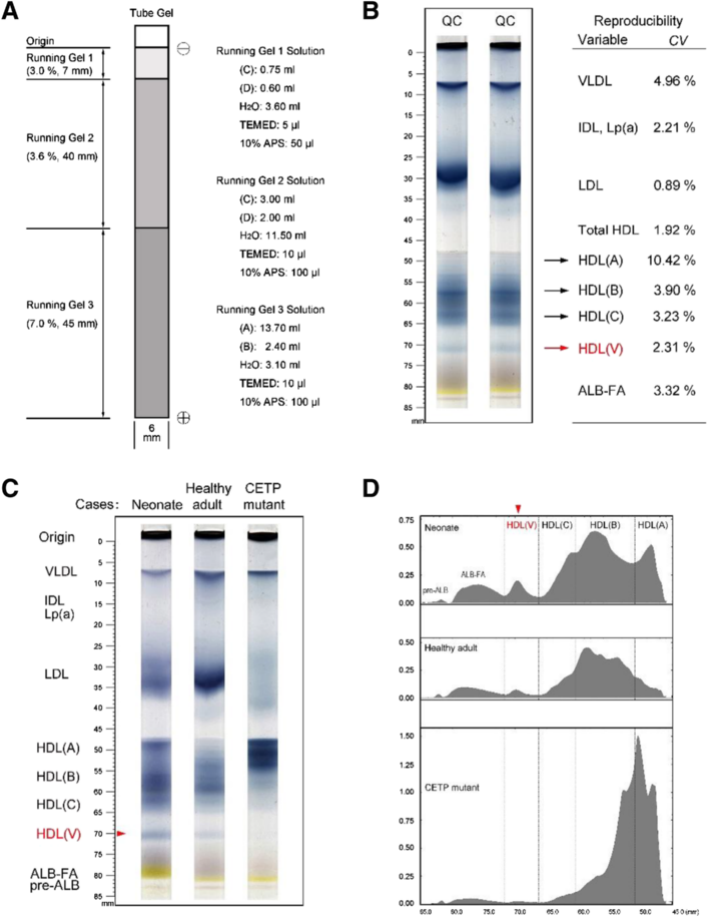
Native PAGE system to separate HDL subclasses. **a** Diagram depicting gel preparation in our new system. Electrophoresis proceeded in the downward direction. Solutions A–D are described in Methods. TEMED, N,N,N’,N’-tetramethylethylenediamine; APS, ammonium persulfate. **b** Tube-gel PAGE of human serum. HDL (V) was indicated in red. CV, interassay coefficient of variation; QC, quality control. **c** Samples were from: a normal neonate, a normal adult, a patient with acute myocardial infarction (AMI), and an adult carrying a *CETP* mutation. HDL (V) was indicated in red. **d** Representative densitometric scans

We used the following six solutions (also see Fig. 1a). 1) Solution A: Acrylamide (9.60 g; Fluca) and 0.25 g N,N’-methylenebisacrylamide (Fluca) in deionized water to 100 ml final volume. 2) Solution B: Tris (18.30 g) to 24 ml of 1 N HCl and diluted to 100 ml final volume with deionized water. 3) Solution C: Acrylamide (19.60 g) and 0.40 g N,N’-methylenebisacrylamide in deionized water to 100 ml final volume. 4) Solution D: Tris (6.06 g) and 1.17 g EDTA-Na_2_ in deionized water to 100 ml final volume. 5) Staining solution: Sudan Black-B (0.125 g; Sigma) to 25 ml isopropanol:ethylene glycol (4:1, V/V), mixed well, and incubated in a water bath at 37 °C overnight. 6) Running buffer: Tris (6.0 g) and 28.8 g glycine in deionized water to 1000 ml final volume, and then diluted 10-fold with deionized water before use.

The gradient gels in 6 × 100 mm glass tubes were prepared as indicated in Fig. 1a. Each tube gel comprised three layers differing in polyacrylamide concentration (7.0, 3.6, and 3.0 %) and height (45 mm, 40 mm, and 7 mm, respectively). Each gel tube was inserted vertically into an electrophoretic cell (DYY-III 27B Model, Liuyi Inc., Beijing). The upper and lower chambers of the electrophoretic cell contained ~400 ml and ~600 ml running buffer, respectively. Serum (100 μl) was mixed with 10 μl of staining solution, and 50 μl was loaded on the top of the tube gel; electrophoresis was carried out for 2.5 h at 100 V. After electrophoresis, each tube was subjected to densitometry (604 nm; Model CS-9301, Shimadzu, Inc. Japan). The sum of the peak areas was taken as the total amount of blood lipids, and the percentage of each lipoprotein fraction was calculated automatically by the densitometer.

### A new two- dimensional gel electrophoresis system

**Fig. 2:**
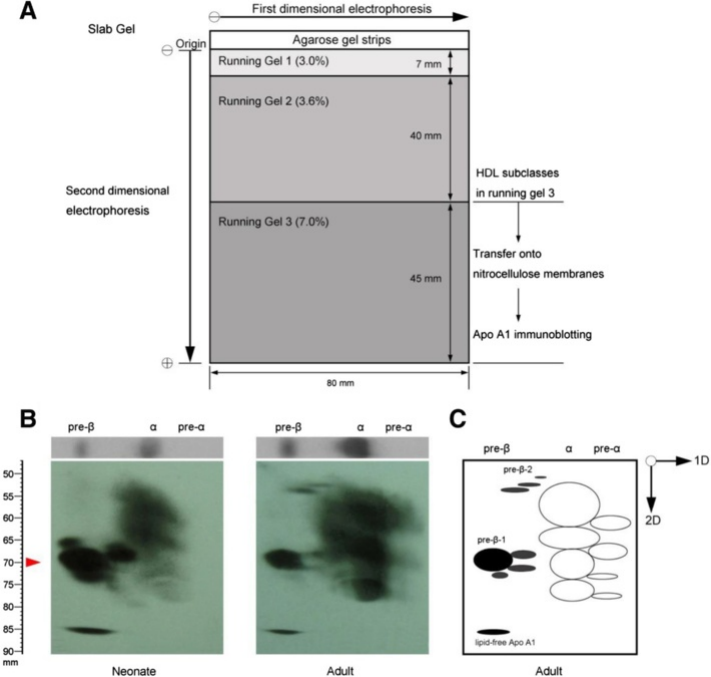
Distribution of apoA-I in human serum determined by nondenaturing two-dimensional nonlinear gradient gel electrophoresis. **a** Schematic diagram of the gel system. **b** Human serum (4 μl) from each of a neonate and adult was subjected to electrophoresis on a 0.75 % agarose gel (first dimension) for 1 h. The lane was then excised and placed on a nonlinear gradient polyacrylamide slab gel (second dimension) and subjected to electrophoresis for 3 h. Serum proteins were transferred to a nitrocellulose membrane, and human apoA-I was visualized with goat anti-human apoA-I. The red arrowhead indicates the distance of particle migration in the electrophoresis. **c** Diagram showing HDL subspecies (in adults) isolated by two-dimensional gel electrophoresis

Serum (4 μl) was applied to a 0.75 % agarose gel (2 mm thick) and subjected to electrophoresis with a Sub-Cell® GT Cell apparatus (Bio-Rad) in a buffer containing 25 mM Tris (pH 8.6) at 100 V for 1.5 h. Gel strips were then excised and placed on top of nonlinear gradient slab gels. Slab gels were prepared as shown in Fig. 2a and had the same gradient setting as tube gels (Fig. 1a). The second-dimension electrophoresis was carried out in an XCell SureLock™ Mini-Cell apparatus (Life Technologies) in a buffer containing 5 mM Tris and 38.4 mM glycine (pH 8.4) at 100 V for 3 h. The separated proteins were transferred to a nitrocellulose membrane. Immunoblotting for apoA-I was performed using polyclonal anti-human apoA-I (Abcam). Horseradish peroxidase–conjugated rabbit polyclonal anti-goat lgG (Novus Biologicals) was used as a secondary antibody. The SuperSignal Westen detection Kit (Pierce) was used for detection.

### Ultracentrifugation

Human serum (6 ml) was adjusted to 1.30 g/ml by adding 2.93 g of KBr. The sample was then added into a 28 ml ultracentrifuge (polyallomer) tube and overlaid with KBr solution (1.063 g/ml) till full. Ultracentrifugation was performed (Rotor: 50.2Ti) with a Beckman L8-M at 40,000 rpm at 10 °C for 10 h. Fractions (1 ml of each) were collected with a long needle from the bottom to top. The density of each fraction was weighed (800 μl was weighed on a scale, Explorer, OHAUS). Each fraction was desalted and concentrated through centricon filters (Millipore) to a final volume of 0.5 ml. These samples were then used for tube gel and two-dimensional gel electrophoresis.

### Electron microscopy

The HDL subclasses were eluted from gels, dialyzed against a solution of 0.4 % NH_4_HCO_3_, and then processed as described [24]. An aliquot of lipoprotein solution was mixed with an equal volume of 2 % sodium phosphotungstate (pH 7.4). A small droplet was applied to a Formvar-grid, where it remained for 30 s. The size and morphology of lipoprotein particles were immediately observed under a transmission electron microscope (JEM-1200EX, Japan) operating at 80 KV. Micrographs were recorded at instrument magnifications of 100 K. The diameter of 100 scattered particles in each lipoprotein fraction was measured using the software of Image-Pro Plus 6.0 (Media Cybernetics, Inc. USA).

### Human study population

The study complied with the Declaration of Helsinki, and was approved by the hospital ethical review board (Zhongshan Hospital, Fudan University, Shanghai, China). A total of 102 subjects underwent diagnostic coronary angiography in Zhongshan Hospital, Fudan University were included in this study, among which, 56 had no stenosis of coronary artery and 46 were diagnosed as CAD, which was predefined as the presence of a luminal diameter stenosis ≥50 % in at least 1 major coronary artery territory (left anterior descending, left circumflex or right coronary artery or their major branches). The severity of the coronary artery stenosis was evaluated by Gensini score [25], which is computed by assigning a severity score to each coronary stenosis according to the degree of luminal narrowing and its geographic importance. Reductions in the lumen diameter of 25 %, 50 %, 75 %, 90 %, 99 % and complete occlusion were given score of 1, 2, 4, 8, 16 and 32, respectively. Each principal vascular segment was assigned a multiplier in accordance with the functional significance of the myocardial area supplied by that segment, that is, the LM was assigned the significant multiplier × 5; the proximal segment of the LAD × 2.5; the proximal segment of the LCX × 2.5; the mid segment of the LAD × 1.5; the RCA, the distal segment of the LAD, the posterolateral artery, and the obtuse marginal artery × 1; and others × 0.5. Diabetes mellitus (DM) was diagnosed by clinical history, use of hypoglycemic medications or a fasting blood sugar level >7.0 mmol/L (125 mg/dL); hypertension was defined in patients receiving antihypertensive treatment or with known diagnosis of hypertension (blood pressure ≥140/90 mmHg).

### Statistical analysis

Continuous variables were reported as means ± standard deviation (SD). Comparisons among more than two groups were performed by analysis of variance (ANOVA), followed by least significant difference (LSD) test for multiple comparisons. The Pearson’s *χ*2 test was used to assess statistical differences in dichotomous and categorical variables. Univariate linear regression analysis was used to determine correlation between Gensini score and HDL-(V) (preβ1-HDL) levels. Attempts were also made to predict CAD by multivariate logistic regression analysis, using CAD as the dependent variable, and age, gender, BMI, smoking history, hypertension, DM, HDL-(V) (preβ1-HDL), HDL-(A), HDL-(B), and HDL-(C) levels as independent variables. To further investigate the relationships between Gensini score and clinical characteristics or laboratory parameters, multivariate linear regression analysis was performed by using Gensini score as the dependent variable, and age, gender, BMI, smoking history, hypertension, DM, HDL-(A), HDL-(B), HDL-(C), and HDL-(V) (preβ1-HDL) levels as independent variables. *P* values < 0.05 were considered statistically significant. All analyses were done using SPSS version 16 analytical software (SPSS Inc., Chicago, IL).

### Biochemical detection

Blood lipids were determined according to standard procedures [26] in the clinical laboratory of Zhongshan Hospital. Lipid concentrations were measured on a Hitachi 911 automatic analyzer, using reagents from Roche Diagnostics. Cholesterol and triglyceride concentrations were determined enzymatically using CHOD-PAP and lipase/GPO/PAP methods, respectively. HDL-cholesterol (HDL-C) concentration was measured with the phosphotungstic acid and MgCl_2_ precipitation approach. LDL-cholesterol (LDL-C) was measured by a direct method, not calculated. The levels of apoAI, apoB, apoE, and Lipoprotein (a) [Lp (a)] were determined by immunoturbidimetric assays.

### Other human samples

Three *CETP* mutant (with G → A in the splice donor site of intron 14 [27]) serums were a gift from Dr. Akihiro Inazu, Department of Clinical Laboratory Science, Kanazawa.

Six Tangier disease serums were from Division of Translational Medicine and Human Genetics, Perelman School of Medicine, University of Pennsylvania.

## Results

The makeup of the native polyacrylamide gels that we developed is shown in Fig. 1a. Non-gradient polyacrylamide gels of 3.0, 3.6, and 7.0 % acrylamide were used for VLDL, LDL, and HDL separation, respectively. Moreover, this system could separate HDL (HDL total) into four fractions denoted here as HDL-(A), (B), (C), and (V) (Fig. 1b). The precision of the assays was established by carrying out ten assays for each of the HDL subclasses in a pooled human adult serum sample. The interassay coefficient of variation for each HDL subclass is shown in Fig. 1b.

We next sought to determine the distribution of these HDL subclasses in different human serum samples. We found that neonates had significantly higher HDL (A) than healthy adults and HDL (B) was the predominant HDL particle in healthy adults. Subjects carrying a *CETP* mutation had much higher HDL (A) levels than all the tested samples (Fig. 1c, d). Importantly, we found that our system could quantitatively separate HDL-(V) from the other subclasses. The order of its abundance was as follows: neonates > healthy adults > persons with a *CETP* mutation.

To characterize these HDL subclasses, we created a new system for two-dimensional gel electrophoresis. All serum lipoproteins were first separated by agarose gel electrophoresis (Fig. 2a). The gel strips were then excised and placed on top of a nonlinear gradient slab gel. As shown in Fig. 2b, this new system yielded patterns comparable with those reported by Francone et al. [17]. We also noticed that human adults had much higher α-HDL and preβ2-HDL compared with neonates, whereas neonates had not only higher preβ1-HDL but also more preβ1-HDL subspecies (Fig. 2b, c).

**Fig. 3:**
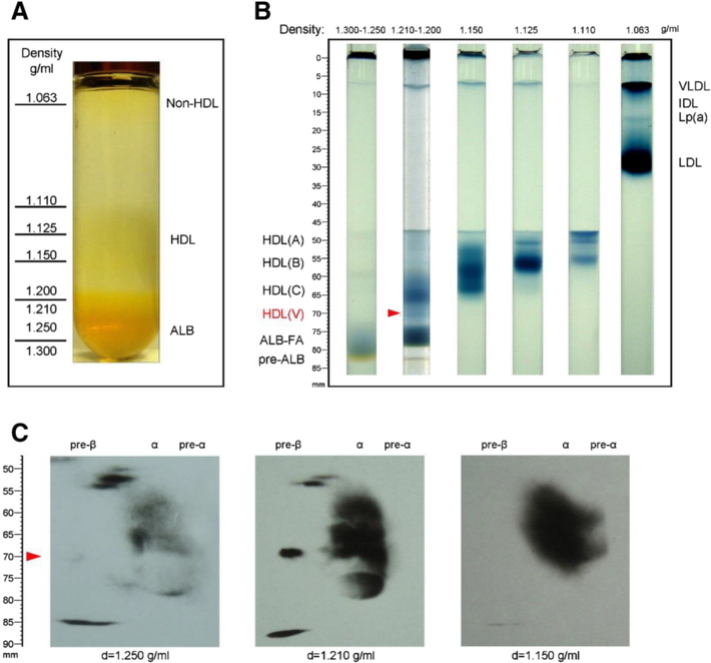
HDL (V) is preβ1-HDL. To prove that HDL-(V) is preβ1-HDL, we isolated different HDL fractions by ultracentrifugation. **a** Isolation of non-HDL and HDL particles using ultracentrifugation. **b** Each fraction was subjected to our new PAGE system. The red arrowhead indicates HDL (V). **c** Each fraction was subjected to our new two-dimensional gel electrophoresis and then immunoblotted with anti-apoA-I. HDL-(V) exhibited an electrophoretic pattern identical to that preβ1-HDL

To further characterize HDL particles, we isolated them by ultracentrifugation (Fig. 3a) and then subjected the Sudan Black pre-stained particles (with different densities) to electrophoresis on our native PAGE gel. We found that HDL-(V), which was well separated from the other HDLs, had the highest density (Fig. 3b). Two-dimensional gel electrophoresis showed that HDL-(V) is, in fact, preβ1-HDL [17], which had a density about 1.21 g/ml (Fig. 3c).

**Fig. 4:**
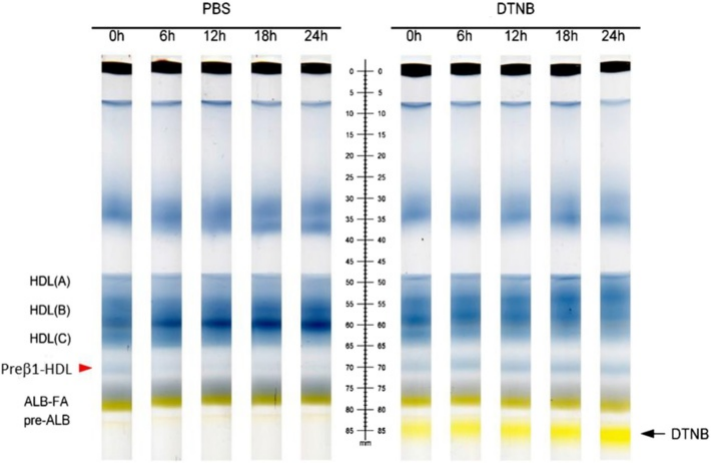
LCAT inhibition blocks preβ1-HDL conversion. Human serum was treated with or without DTNB (1.8 mM) and then pre-stained with Sudan Black. The sample was run on the gel

To further evaluate that HDL-(V) is preβ1-HDL, we treated human serum with DTNB (1 mM) to inhibit LCAT [28] and then ran the pre-stained serum on the native PAGE gel. We found that without DTNB treatment, HDL-(V) gradually disappeared during the 6-h incubation, indicating a LCAT-mediated process for HDL maturation, from preβ1-HDL (nascent HDL) to matured HDL. However, DTNB treatment could prevent this process (Fig. 4).

**Fig. 5:**
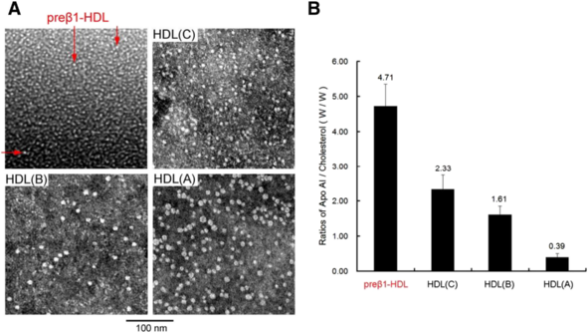
Characterization of HDL subclasses. **a** Size and shape of particles of the HDL subclasses as viewed with electron microscopy. The mean diameter of the particles of preβ1-HDL (red arrows), HDL-(C), HDL-(B), and HDL-(A) was 5 nm, 7 nm, 9 nm, and 11 nm, respectively. The bar indicates 100 nm. **b** ApoA-I/cholesterol ratios in 9 separated samples

We next determined the particle size of the isolated HDL subclasses using electron microscopy with a negative-staining approach. A scattered pattern of isolated HDL particles was observed at 100,000× magnification (Fig. 5a). All particles were spheroidal. The preβ1-HDLs were visualized as small dot-like particles rather than disc-like ones in which they exhibited a weak contrast. The mean diameter of the HDL particles was as follows: (A) = 11 nm, (B) = 9 nm, (C) = 7 nm, and preβ1-HDL = 5 nm. We also measured apoA-I and cholesterol levels in each fraction and found that preβ1-HDL had the highest apoA-I/cholesterol ratio, indicating that although it is lipid poor it is not lipid free (Fig. 5b). This was consistent with the results obtained from our native PAGE because the lipids in each HDL were pre-stained with Sudan Black (Fig. 1b, c).

**Fig. 6:**
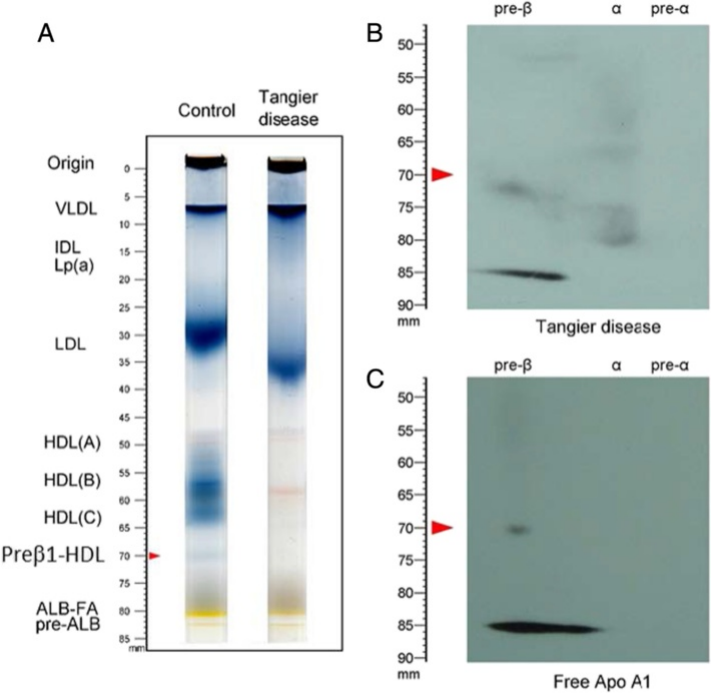
A two-dimensional gel system cannot precisely quantify preβ1-HDL. **a** Tangier patient (pooled from 5 subjects) and control serum running on the native PAGE system as shown in Fig. 1. **b** Tangier sample running on the two-dimensional gel system. **c** purified apoA-I (200 ng) running on the two-dimensional gel system

We analyzed Tangier patient pre-stained serum (pooled from 5 samples) on our native PAGE gel and found that there was no preβ1-HDL (Fig. 6a). However, when we ran the same sample on our two-dimensional gel, we found there was a preβ1-HDL-like spot (Fig. 6b), suggesting that the two-dimensional gel cannot distinguish real preβ1-HDL particles and preβ1-HDL-like apoA-I aggregates. To see the lipid-free apoA-I aggregate does exist, we ran purified apoA-I (Sigma) on the two-dimensional gel and found the apoA-I sample contains preβ1-HDL-like/lipid-free apoA-I aggregates (Fig. 6c). Thus, the two-dimensional gel system cannot precisely quantify preβ1-HDL in human serum.

**Fig. 7:**
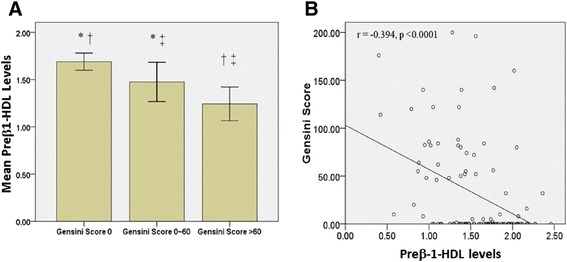
Comparisons of preβ1-HDL levels in patients having different Gensini score. **a** There were 56, 22 and 24 individuals having Gensini score 0, 0 ~ 60 and >60, respectively. Mean preβ1-HDL levels among the 3 groups were significant different by ANOVA test (*p* < 0.0001). By LSD test, there were significant different preβ1-HDL levels between Gensini score 0 and 0 ~ 60 (* *p* = 0.0032), 0 and >60 († *p* <0.0001), and 0 ~ 60 and >60 (‡ *p* = 0.047). **b** Univariate linear regression analysis showed a negative correlation between Gensini score and preβ1-HDL levels (*r* = −0.394, *p* < 0.0001)

Further, we utilized our one-dimensional method to measure preβ1-HDL levels in 102 human subjects. Concerning that Gensini sore > 60 was related to severe coronary artery stenosis and poor prognosis [25], all these 102 subjects were divided into three groups according to Gensini score (group 1, Gensini score = 0, *n* = 56; group 2, Gensini score 0 ~ 60, *n* = 22; group 3, Gensini score > 60, *n* = 24). Clinical characteristics and parameters of the three groups and significant differences are shown in Table 1. Smoking history, levels of HDL-C, preβ1-HDL, HDL-(A), HDL-(B), and IDL were significantly different in the three groups. Further multiple comparisons by LSD test showed significant difference between every two groups in smoking history, levels of preβ1-HDL, and IDL (Table 1, Fig. 7a).

**Table 1:** Group comparisons for different variables in different Gensini score groups: Continuous variables are given as means ± SD and evaluated using analysis of variance (ANOVA), followed by least significant difference (LSD) test for multiple comparisons. Dichotomous and categorical variables are given as percentages and and compared using Pearson’s *χ* ^2^ test

Variable	Group 1 (Gensini score = 0, *n* = 56)	Group 2 (Gensini score = 0 ~ 60, *n* = 22)	Group 3 (Gensini score >60, *n* = 24)	*p* value
Male (N%)	43 (76.8 %)	18 (81.8 %)	18 (75.0 %)	0.845
Age (years)	59.75 ± 12.67	57.82 ± 13.83	65.83 ± 9.70	0.062
BMI (kg/m^2^)	23.85 ± 4.21	25.58 ± 3.18	23.39 ± 3.30	0.122
Smoking (N%)	3 (18.8 %)	15 (68.2 %)	13 (54.2 %)	0.009
				0.003 (Group 1 vs. 2)
				0.025 (Group 1 vs. 3)
				0.331 (Group 2 vs. 3)
Hypertension (N%)	9 (56.2 %)	12 (54.5 %)	14 (58.3 %)	0.967
DM (N%)	2 (12.5 %)	4 (18.2 %)	6 (25.0 %)	0.609
TC (mmol/L)	4.35 ± 0.74	4.20 ± 1.14	4.52 ± 1.10	0.507
TG (mmol/L)	1.29 ± 0.56	1.62 ± 0.93	1.71 ± 1.27	0.077
HDL-C (mmol/L)	1.39 ± 0.31	1.09 ± 0.19	1.14 ± 0.29	<0.0001
				<0.0001 (Group 1 vs. 2)
				<0.0001 (Group 1 vs. 3)
				0.507 (Group 2 vs. 3)
LDL-C (mmol/L)	2.39 ± 0.58	2.38 ± 0.90	2.62 ± 0.84	0.381
ApoAI (mg/mL)	0.97 ± 0.13	0.98 ± 0.15	1.08 ± 0.23	0.078
ApoB (mg/mL)	0.70 ± 0.19	0.82 ± 0.29	0.88 ± 0.30	0.143
ApoE (mg/L)	43.52 ± 12.78	46.05 ± 17.38	51.87 ± 20.65	0.309
Lp (a) (mg/L)	187.40 ± 182.46	191.16 ± 179.62	188.22 ± 191.16	0.998
HDL-(A) (%)	1.94 ± 0.77	1.48 ± 0.64	1.81 ± 0.71	0.048
				0.014 (Group 1 vs. 2)
				0.451 (Group 1 vs. 3)
				0.135 (Group 2 vs. 3)
HDL-(B) (%)	19.11 ± 6.73	13.70 ± 4.77	15.46 ± 6.24	0.001
				0.011 (Group 1 vs. 2)
				0.018 (Group 1 vs. 3)
				0.343 (Group 2 vs. 3)
HDL-(C) (%)	10.45 ± 2.36	11.15 ± 2.97	9.69 ± 2.89	0.171
Preβ1-HDL (%)	1.69 ± 0.34	1.48 ± 0.47	1.24 ± 0.42	<0.0001
				0.032 (Group 1 vs. 2)
				<0.0001 (Group 1 vs. 3)
				0.047 (Group 2 vs. 3)
LDL (%)	41.90 ± 5.88	42.81 ± 7.02	44.34 ± 5.92	0.268
IDL (%)	3.58 ± 1.06	5.14 ± 1.71	6.02 ± 2.28	<0.0001
				<0.0001 (Group 1 vs. 2)
				<0.0001 (Group 1 vs. 3)
				0.006 (Group 2 vs. 3)
VLDL (%)	16.37 ± 6.10	20.12 ± 8.38	17.95 ± 8.24	0.116

*BMI* body mass index, *DM* diabetes mellitus, *TC* total cholesterol, *TG* triglyceride, *HDL-C* high density lipoprotein cholesterol, *LDL-C* low density lipoprotein cholesterol, *ApoAI* apolipoprotein A, *ApoB* apolipoprotein B, *ApoE* apolipoprotein E, *Lp (a)* Lipoprotein (a). The sum of the peak areas (after scanning see Fig. 1) was taken as the total amount of plasma lipoproteins, and the percentage (%) of each lipoprotein fraction was calculated automatically by the densitometer

By multivariate logistic regression analysis (Table 2), both smoking history and preβ1-HDL (aOR = 0.13, *p* = 0.05) levels in CAD patients were found to be independently associated with CAD.

**Table 2:** Logistic regression predictors of CAD: Logistic regression analysis was performed by using CAD as the dependent variable, and age, gender, BMI, smoking history, hypertension, DM, preβ1-HDL, HDL-(A), HDL-(B), and HDL-(C) as independent variables

Variables	*p* value	aOR	95.0 % C.I. for aOR
Age (years)	0.31	1.04	(0.96-1.14)
Male gender	0.43	1.93	(0.37-9.99)
BMI (kg/m^2^)	0.27	1.14	(0.90-1.45)
Smoking	0.03	9.77	(1.28-74.64)
Hypertension	0.97	1.03	(0.17-6.22)
DM	0.95	1.08	(0.10-11.67)
HDL-(A)	0.10	4.75	(0.73-30.86)
HDL-(B)	0.11	0.84	(0.68-1.04)
HDL-(C)	0.52	0.91	(0.68-1.22)
Preβ1-HDL	0.05	0.13	(0.02-1.02)

To study the relationship between the severity of coronary artery stenosis and preβ1-HDL levels, univariate linear regression analysis was performed to show a negative correlation between Gensini score and preβ1-HDL levels (*r* = −0.394, *p* < 0.0001, Fig. 7b). Further, using multivariate linear regression analysis, DM (B = 43.57, *p* = 0.01) and preβ1-HDL levels (B = −37.33, *p* = 0.01) were independently associated with Gensini score after adjusted for age, gender, BMI, hypertension, HDL-(A), HDL-(B), and HDL-(C) levels (Table 3). Thus, preβ1-HDL levels are independent predictors of higher Gensini score.

**Table 3:** Predictors of Gensini score by multivariate linear regression: Multivariate linear regression analysis was performed by using Gensini score as the dependent variable, and age, gender, BMI, smoking history, hypertension, DM, HDL-(A), HDL-(B), HDL-(C), and preβ1-HDL levels as independent variables

Variables	*p* value	B	95 % confidence interval for B
Age (years)	0.10	1.10	(−0.23-2.43)
Male gender	0.62	8.22	(−24.80-41.24)
BMI (kg/m^2^)	0.25	−2.39	(−6.46-1.69)
Smoking	0.44	11.74	(−18.77-42.25)
Hypertension	0.58	8.60	(−22.75-39.95)
DM	0.01	43.57	(10.06-77.07)
HDL-(A)	0.31	13.06	(−12.65-38.76)
HDL-(B)	0.32	−1.73	(−5.20-1.74)
HDL-(C)	0.50	−1.86	(−7.09-3.38)
Preβ1-HDL	0.01	−37.33	(−66.61- -8.05)

## Discussion

In this study, we 1) developed a new native PAGE system which quantitatively separates a smaller HDL particles from other HDL particles; 2) demonstrated that this HDL fraction is preβ1-HDL, which can be converted to other HDLs by LCAT; 3) found that preβ1-HDL particles thus isolated are spheroidal with the highest apoA-I/cholesterol ratio and highest density (≥1.21 g/ml), compared with all other HDL particles; 4) found that neonates have significantly higher and more complex preβ-HDLs than adults; 5) found that Tangier patients and *CETP* mutation have no detectable preβ-HDLs; 6) found that the existing two-dimensional gel system cannot precisely quantify preβ1-HDL in human serum; and 7) found that preβ1-HDL is significantly related to the severity of coronary artery stenosis in a negative manner.

One of the key accomplishments of this study is that we established a simple and fast method for preβ1-HDL quantification. It has been reported that HDL subclasses can be defined by either of two gradient PAGE systems [14, 15], but the resolution of neither system is ideal. The novelty of our system is that we assemble three different polyacrylamide gels (3.0, 3.6, and 7.0 % acrylamide) to separate VLDL, LDL, and HDL and also to separate HDL subclasses in a single-step electrophoresis. The biggest difference between our native PAGE system and previous systems is that we can clearly resolve HDL (V), i.e. preβ1-HDL (Fig. 1b), whereas other systems cannot detect this fraction [14, 15]. Moreover, our system has a small interassay coefficient of variation (Fig. 1b).

The second key accomplishment of this study is that we established a new system for HDL two-dimensional gel electrophoresis. It has been reported that HDL subclasses can be defined on two-dimensional systems (agarose/linear gradient polyacrylamide gel) [17, 20]. Based on these reported systems, we established our own agarose/nonlinear gradient PAGE system applicable to any typical research or clinical laboratory (Fig. 2).

Preβ1-HDL has unique characteristics. We found that preβ1-HDL particles isolated with our PAGE system have higher density (≥1.21 g/ml; Fig. 3a, b) than other HDLs, and this was confirmed by two-dimensional gels (Fig. 3c). With respect to particle structure, many studies have postulated that preβ1-HDL is discoidal [29–32], but no report has shown this directly. Discoidal HDL, having an electrophoretic mobility like that of preβ1-HDL, can be synthesized in vitro to serve as a model for plasma preβ-HDL [31, 33]. Our electron microscopy analysis revealed no discoidal particles but rather only spheroidal particles (Fig. 5a), similar to a previous report [23]. Moreover, we also found that preβ1-HDL contains lipids (Figs. 1b, c and 5b), which is different from the previous report [23].

Plasma preβ1-HDL plays an important role in cholesterol efflux, but it remains controversial whether preβ1-HDL is the substrate or the first product of ABCA1-mediated cholesterol efflux [34, 35]. It has been reported that “preβ1-HDL-like” lipid-poor apoA-1 or nascent HDL is formed when lipid-free apoA-1 retrieves phospholipids and cholesterol from cells mediated by ABCA1 in experiments using various culture cells [36, 37]. Thus, the levels of preβ1-HDL should be an indicator of the capacity for HDL production and reverse cholesterol transport, and preβ1-HDL should be expected to have an anti-atherogenic function. However, the results from literatures suggest that an elevated level of preβ1-HDL is a risk factor for CAD [19–21]. This could be due the two-dimensional electrophoresis cannot separate preβ1-HDL from the lipid-free apoA-I. Our new two-dimensional gel system not only separated preβ1-HDL from the lipid-free apoA-I, but also demonstrated a complex pattern of preβ1-HDL, which was not reported before (Fig. 2b, c).

Two-dimensional electrophoresis system is not suitable for preβ1-HDL measurement. It has been reported that patients with Tangier disease have preβ1-HDLs, but not mature HDL [22]. However, when we carefully re-checked the two-dimensional electrophoresis gel presented in current study, the homozygous patient might only have lipid-free apoA-I (as recently suggested by Miyazaki et al. [23]). Importantly, we found that samples from subjects with Tangier disease do not contain preβ1-HDLs when using our one-dimensional native PAGE gel (Fig. 6a), however, when we examined the sample on our two-dimensional gel system, we did find a preβ1-HDL-like spot (Fig. 6b), probably consisting in preβ1-HDL-like/lipid-free apoA-I aggregates (Fig. 6c). Thus, the two-dimensional gel system cannot precisely quantify preβ1-HDL in human serum. It is reasonable to infer that Tangier patients have only lipid-free apoA-I (which could be monomer and polymer or aggregate) but have no preβ1-HDL, since they have no ABCA1-mediated cholesterol efflux and preβ1-HDL cannot be formed, thus, no apoA-I-containing mature HDL can be produced.

Using our new one-dimensional PAGE system, we can precisely separate preβ1-HDL from the rest of HDL. This relatively simple method helped us to re-evaluate the relationship between preβ1-HDL and CAD. We found that preβ1-HDL levels are independently and negatively associated with the severity of the coronary artery stenosis (Table 3, Fig. 7a, b).

Interestingly, we also found that subjects carrying a *CETP* mutation have not only larger HDLs but also no detectible preβ1-HDL with our system (Fig. 1c). This preβ1-HDL deficiency may be one of the reasons why CETP inhibitors, to date, do not work in the expected direction, i.e., to reduce the incidence of cardiovascular diseases.

Another interesting observation from this study is that neonates have much higher and more complex preβ1-HDLs than adults, indicating that there are age-related changes in the characteristics of circulating preβ1-HDLs. If we can slow down such changes, we may find a new way to slow down the development of atherosclerosis.

We also observed some interesting phenomena, few of which have been reported before (Table 1). We found that 1) in healthy adults, HDL-(B) is the major HDL particle, and 2) neonates have a dramatically different pattern of HDL subclasses compared with healthy adults. Although we do not know the physiological relevance of these differences, we now have, at least, a way to view them.

There are some limitations of our system. The preparation of tube gels is time consuming; however, this process can be standardized. Further optimization is needed to improve the resolution of HDL-(A), −(B), and -(C). Regardless, the simple method for preβ1-HDL quantification we developed is suitable for clinical applications and may also be appropriate for screening potential drugs that may increase the level of circulating preβ1-HDLs.

## Conclusion

We established a novel and simple method for human serum preβ1-HDL quantification. We found that human lower preβ1-HDL is an independent predictor for severer coronary artery stenosis.

## Abbreviations

CETP: cholesteryl ester transfer protein
HDL: high density lipoprotein
LCAT: lecithin-cholesterol acyltransferase
PAGE: polyacrylamide gel electrophoresis
